# Simultaneous quantification of myocardial and blood flow velocities based on duplex mode ultrasound imaging

**DOI:** 10.1186/1475-925X-12-107

**Published:** 2013-10-16

**Authors:** Christer Grönlund, Kenji Claesson, Jan D’hooge, Michael Y Henein, Per Lindqvist

**Affiliations:** 1 Department of Biomedical Engineering – R&D, Radiation Sciences, Umeå University, Umeå 90185, Sweden; 2 Centre for Biomedical Engineering and Physics (CMTF), Umeå University, Umeå, Sweden; 3 Department of Cardiovascular sciences, KU Leuven, Leuven, Belgium; 4 Heart center and Department of Public Health and Clinical Medicine, Umeå University, Umeå, Sweden

**Keywords:** Ultrasound, Heart, Simultaneous, Echocardiography, Blood flow, Myocardial, Velocity, Low frame rate, Duplex

## Abstract

**Background:**

Ultrasound imaging of the heart is a commonly used clinical tool to assess cardiac function. The basis for this analysis is the quantification of cardiac blood flow and myocardial velocities. These are typically measured using different imaging modes and on different cardiac cycles. However, due to beat-to-beat variations such as irregular heart rhythm and transient events, simultaneous acquisition is preferred. There exists specialized ultrasound systems for this purpose; however, it would be beneficial if this could be achieved using conventional ultrasound systems due to their wide availability. The conventional Duplex mode ultrasound allows simultaneous acquisition, however at a highly reduced spatial and temporal resolution.

**Methods:**

The aim of this work was to present and evaluate the performance of a novel method to recover myocardial tissue velocity using conventional Duplex ultrasound imaging, and to demonstrate its feasibility for the assessment of simultaneous blood flow and myocardial velocity in-vivo. The essence of the method was the estimation of the axial phase shift of robust echogenic structures between subsequent image frames. The performance of the method was evaluated on synthetic tissue mimicking B-mode image sequences at different frame rates (20–60 Hz) and tissue velocities (peak velocities 5-15cm/s), using cardiac deformation and displacement characteristics. The performance was also compared to a standard 2-D speckle tracking technique.

**Results:**

The method had an overall high performance at frame rates above 25 Hz, with less than 15% error of the peak diastolic velocity, and less than 10 ms peak timing error. The method showed superior performance compared to the 2-D tracking technique at frame rates below 50 Hz. The in-vivo quantification of simultaneous blood flow and myocardial tissue velocities verified the echocardiographic patterns and features of healthy subjects and the specific patient group.

**Conclusions:**

A novel myocardial velocity quantification method was presented and high performance at frame rates above 25 Hz was shown. In-vivo quantification of simultaneous myocardial and blood flow velocities was feasible using the proposed method and conventional Duplex mode imaging. We propose that the methodology is suitable for retrospective as well as prospective studies on the mechanics and hemodynamics of the heart.

## Background

### Cardiac ultrasound and beat-to-beat variations

Ultrasound imaging of the heart is a commonly used clinical tool to assess cardiac function. The basis for this analysis is the quantification of cardiac blood flow velocities and myocardial motion. Typically, they are consecutively acquired using separate ultrasound imaging modes, i.e., on different cardiac beats. Thus, they are not measured in a simultaneous fashion, and beat-to-beat variations such as physiological transients (e.g., stress), respiration (load), and heart rate variability is not accounted for [1]. In particular, diagnostic assessment of patients with irregular heart rhythms, atrial fibrillation, and diastolic dysfunction would improve from a simultaneous measurement [2].

### Simultaneous myocardial and blood flow velocity

Recently, techniques for simultaneous acquisition of myocardial motion and blood flow velocity were demonstrated. However, they were based on specialized ultrasound scanner systems with customized imaging modes [2,3]. Thus these techniques are not available to the vast majority of ultrasound users.

On conventional clinical ultrasound systems the Duplex imaging mode achieves a simultaneous acquisition of myocardial tissue and blood flow velocity (Color Doppler mode, CDI). Here, the Doppler mode images are interleaved with B-mode images, and colour-coded on top of the greyscale images. This results in relatively low frame rates (typically 20-40 Hz) of the B-mode sequence; there is a trade-off between CDI and B-mode frame rates (typical setting is 3:1 between CDI frame rate and B-mode frame rate). In addition, the B-mode images comprise less image lines compared to standard B-mode, resulting in a crude 2-D representation of the tissue structures (lower lateral spatial resolution).

Due to the low frame rate and low lateral spatial resolution the speckle pattern decorrelation is very high [4], and the relative lateral and out-of-plane motion of the heart will be significant. As a consequence, traditional tissue velocity estimation using speckle tracking methods [5,6] performs poorly and dedicated software may not allow the estimation of myocardial velocities.

### Aim

The aim of this work was to 1) present and evaluate the performance of a novel method to recover myocardial tissue velocity using conventional duplex ultrasound imaging, and to 2) exemplify its feasibility for the assessment of simultaneous blood flow and myocardial velocity in-vivo.

## Methods

### Myocardial velocity quantification

**Figure 1:**
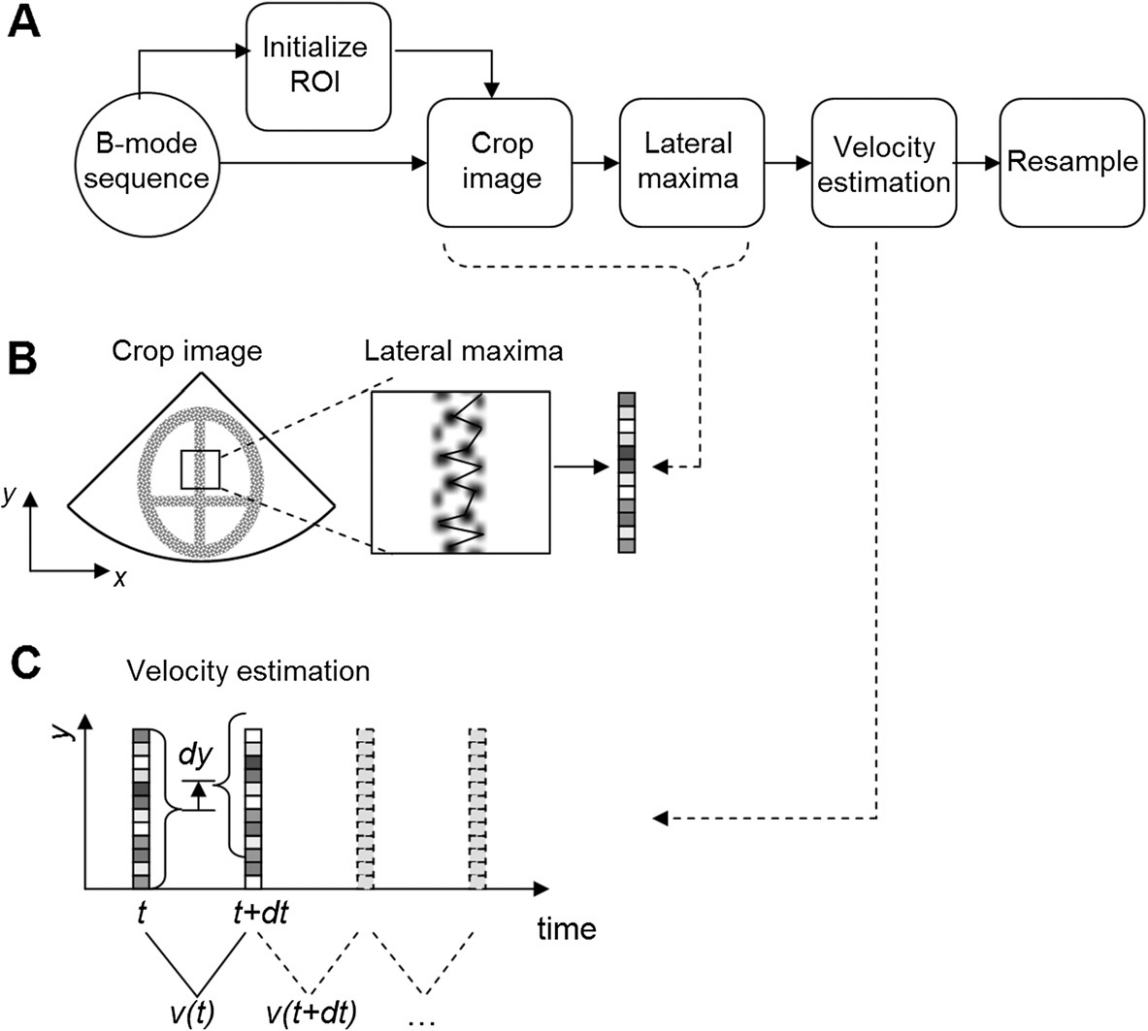
**Illustration of the myocardial tissue velocity quantification procedure. (A)** Block scheme of the procedure including five steps: Initially. a (ROI) was selected. Subsequently, the ROI was used for cropping the images in the sequence, prior to reduction of the 2D B-mode images’ echo-pattern to 1D projections. The tissue velocity was estimated using spatial phase shift and finally the velocity signal was resampled to increase temporal resolution. **(B)** Illustration of ROI selection, cropped image and reduction of image to 1D by taking the lateral echogenic maxima. **(C)** Myocardial velocity was estimated based on the spatial phase shift of the 1D projections between pair-wise consecutive frames. Color of schematic ultrasound images are inverted, where dark area corresponds to an echogenic region.

The method to recover the axial tissue velocity was based on five steps (block scheme of Figure 1A). Initially, a 2-D region-of-interest (ROI) was selected in the image. Next, the 2-D B-mode images were cropped to the size of the ROI (Figure 1B). The cropped image sequence will here be denoted *I*(*y*, *x*, *t*), where *y* is depth (axial), *x* is width (lateral), and *t* is time. Subsequently, the 2-D images were reduced to 1-D representations by taking the laterally most echogenic structures (Figure 1B) as:

(1) $$I'\left(y,t\right)=\underset{x}{\text{max}}\;I\left(y,x,t\right).$$

The motivation for this reduction was that the tissue has a rather poor 2-D image representation due to relatively few image lines. In addition, the most highly reflective pixels have the best signal-to-noise ratio (SNR), and represent larger structures (and not speckle-pattern), thus more likely to be visible between frames at low frame rates and thus more robust to out-of-plane motion.

The axial tissue velocity was estimated based on the spatial phase-shift of pairwise consecutive 1-D tissue profiles (Figure 1C). The spatial phase-shift was calculated using the lag, *n*_*y*_ of the maximum of the cross-correlation function, *R*, as

(2) $$n_{y}\left(t\right)=\underset{y}{\text{argmax}}\;R\left(I'\left(y,t+\text{dt}\right),I'\left(y,t\right)\right).$$

The corresponding tissue velocity was then calculated as

(3) $$v\left(t\right)=n_{y}\left(t\right)\cdot \frac{\text{dy}}{\text{dt}},$$

where *dy* is the axial resolution of the images. To increase the resolution of the estimated velocity, the spatial dimension of the 1-D signals, *I’(y,t)*, were up-sampled by a factor of 20 prior to the cross-correlation calculation.

Finally, the tissue velocity signal, *v(t)*, was resampled at 1000 Hz (from the original 20–60 Hz) using interpolation (cubic splines) in order improve the waveform resemblance and allow estimation of timing of peaks.

### Evaluation of performance of myocardial velocity quantification

**Figure 2:**
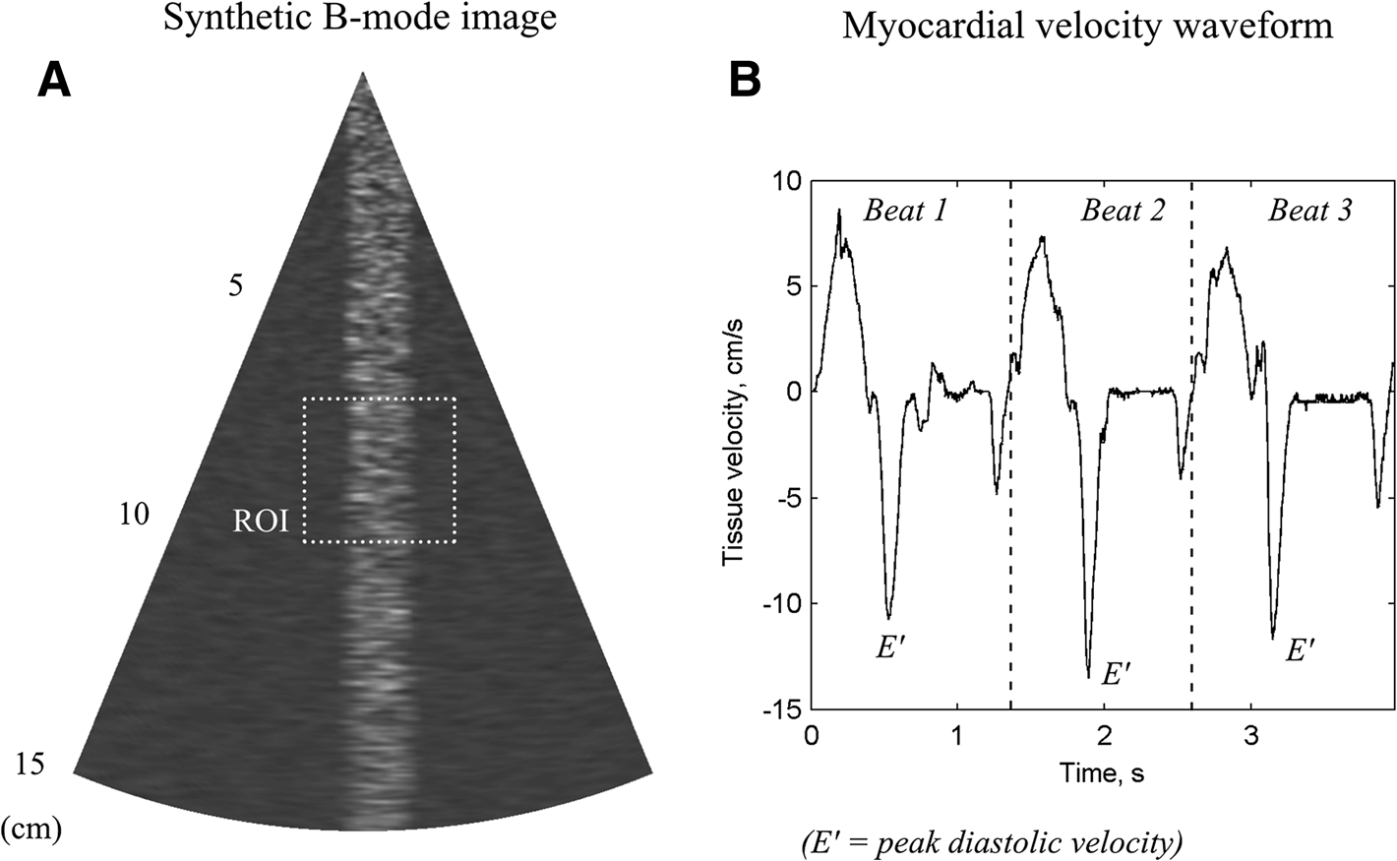
**Example of synthetic image and velocity waveforms. (A)** B-mode sector scan of the synthetic septum cardiac tissue and **(B)** the waveform of authentic myocardial tissue velocity (three beats) used in the evaluation of the performance of the myocardial velocity quantification. Region-of-interest (ROI) used for tissue velocity estimation is shown in **A**.

The performance of the method was evaluated using synthetic B-mode septum tissue mimicking image sequences (see Appendix A for calculation details). Matlab code (version 2011b, Mathworks, Nattick, MA, USA) for generating the data can be downloaded at http://www.vll.se/mt/fou/). In essence, synthetic B-mode image sector scans with 64 lines at 45 degrees field-of-view were generated by sampling image planes of a synthetic 3-D tissue volume (2-D image section shown in Figure 2A) after imposing lateral and out-of-plane motion displacement of the tissue. In addition, the axial displacement was achieved by deforming the synthetic volume in the axial direction. The tissue motion was based on authentic myocardial tissue velocity waveforms (Figure 2B). Ninety-six cardiac-cycles were simulated for combinations of frame rates between 20 to 60 Hz, and diastolic peak velocities at 5, 10 and 15 cm/s. The cardiac cycle tissue waveform for each simulation was randomly selected out of three different ones (Figure 1B). The resulting axial strain was about 6, 12, and 18% for 5, 10 and 15 cm/s velocities, respectively.

**Figure 3:**
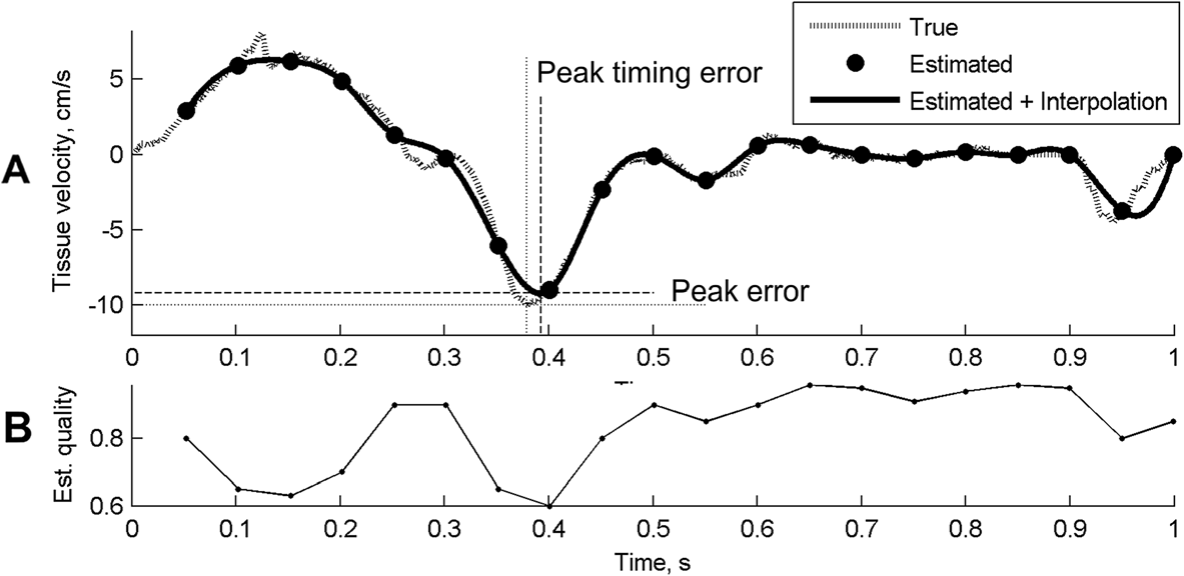
**Measures of performance. (A)** Example of estimated and true myocardial velocity curves from a typical simulation of one cardiac cycle at 20 Hz frame rate. Peak error and peak timing error was calculated as the difference between the true waveform peak amplitude and position, respectively. The velocity estimation quality (cross-correlation peak) is illustrated in **B**.

The performance of the proposed method was assessed using A) the estimation error between the true peak and the estimated peak velocity (E’), B) the timing error between the true and estimated peaks, and C) the cross-correlation value at the E’ (as a measure of estimation quality). See Figure 3A for illustration of measures of performance.

In addition, the performance of the proposed method was compared to a standard 2-D speckle-tracking technique. Two-dimensional cross-correlation was used to calculate the axial spatial lag between pair wise consecutive frames of the cropped image sequence, *I(y,x,t)*. Prior to the 2-D cross-correlation calculation, the axial resolution of the images were up-sampled by a factor 20, and the final tissue velocity signal was re-sampled at 1000 Hz using interpolation (cubic splines). Thus, the velocity estimation resolution and temporal resolution was comparable as with the proposed method.

The methods were compared using two separate sets of simulated signals. The first set featured pure axial displacement of tissue, i.e., no out-of-plane motion and no deformation (so called “best-case” conditions). The second set featured axial deformation as well as out-of-plane motion (so called “worst-case” conditions).

### In-vivo duplex mode acquisition

Two subjects participated in this work; one healthy subject, age 40 years, with no medical history of cardiac disease, and one patient with cardiac disease: biopsy-proved hereditary transthyretin amyloidosis, age 45. Each participant gave a written consent prior to the tests. The test conformed to the declaration of Helsinki and was accepted by the local ethics committee of Umeå University, Sweden.

Ultrasound image sequences where acquired using a Vivid 7 ultrasound scanner with a M4S cardiac probe (GE Medical, Horten, Norway) in Duplex mode (B/CDI). Sector depth, width and location were optimized for maximal frame rate, and allowing simultaneous registration of motion of the septal myocardial wall and the blood flow velocity across the mitral annulus. The frame rate of the CDI and B images were about 100 Hz and 33 Hz, respectively. Image sequences were exported in hdf5 format, with B-mode and CDI-modes as separate image sequences at 8-bit resolution, for offline processing.

### Blood flow velocity quantification

First, the CDI images of the duplex mode where filtered using a 3 × 3 pixel 2-D median filter. Next, the mitral blood flow velocity was extracted from a ROI covering the tip of the open mitral valves. The distribution of blood flow velocities from within this ROI was visualized as a spectral representation including all frames of the entire cardiac beat.

## Results

### Performance of myocardial velocity quantification

Figure 3 presents the estimated and true myocardial velocity curves from a typical simulation of one cardiac cycle. In particular, the improvement of the interpolation on the sampled velocity curve can be seen in Figure 3A, in terms of both peak velocity accuracy and its timing as compared to the true velocity curve.

**Figure 4:**
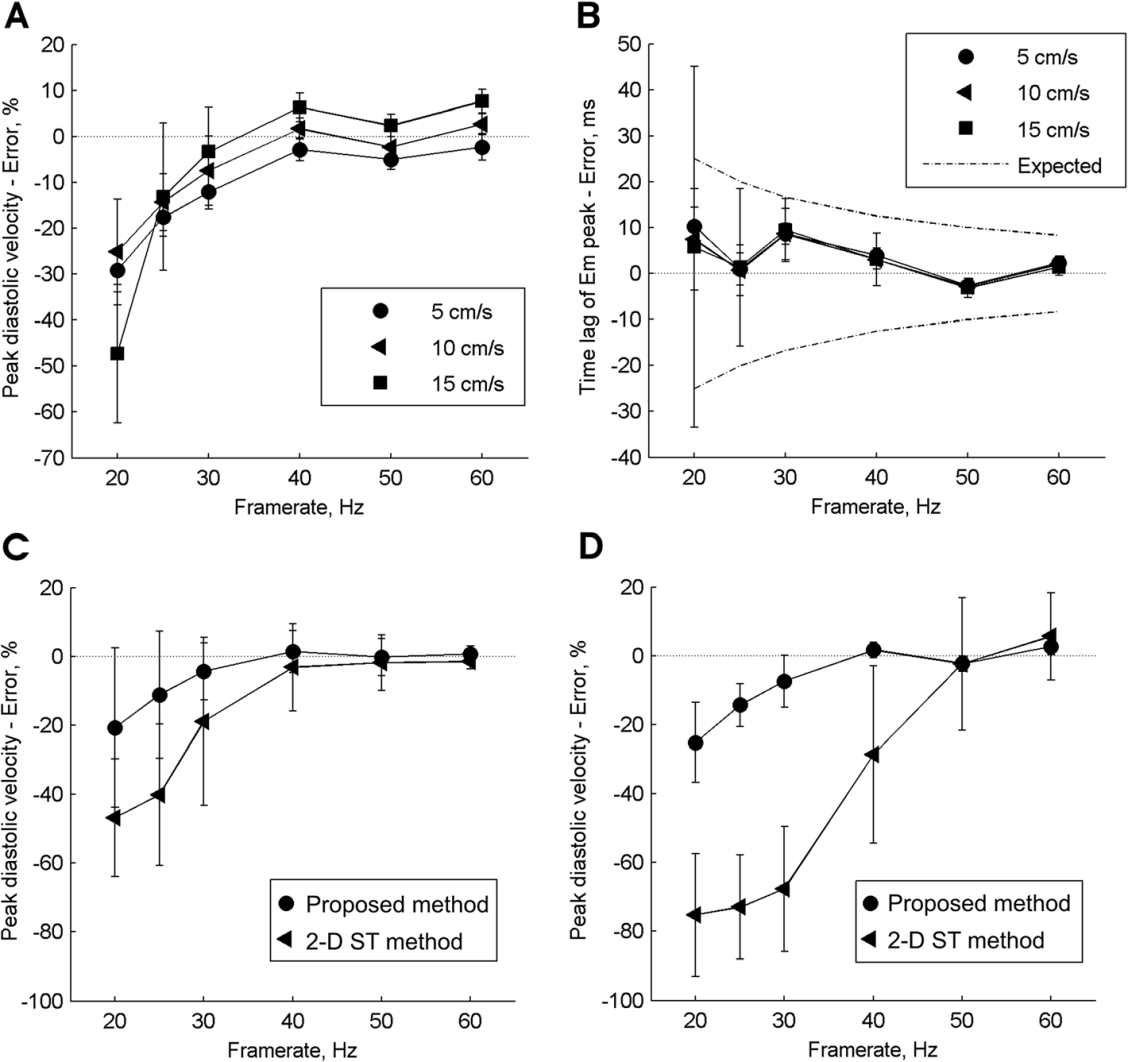
**Performance of myocardial velocity quantification method. A)** The peak error was calculated as the relative difference between the true and estimated velocities. **B)** Peak timing error was the time difference between the true peak position and the estimated peak position. The theoretical limit (dotted line) is half the inverse of frame rate and is the upper limit for the non-interpolated timing error. **C)** Comparison of performance between the proposed method and 2-D speckle tracking method in mild “best case” conditions (no lateral or out-of-plane motion, and only axial displacement of tissue). **D)** Same as in C but in rough “worst case” conditions (including axial deformation, and out-of-plane motion).

The results of the performance evaluation are presented in Figure 4A-B. In general, the performance increased with increasing frame rate and decreasing tissue velocity. When the frame rate was above 25 Hz, the peak velocity error was on average less than -15% (Figure 4A), and the peak timing error was less than 10 ms (Figure 4B). It should be stressed that the theoretical average timing error at 1/(2*frame rate) without waveform interpolation is much higher (illustrated by dotted line in Figure 4B). The estimation quality (cross-correlation values at the estimated peak) increased with increasing frame rate, was in the range 0.5 to 0.9, and was higher than 0.75 for frame rates above 25 Hz.

Figure 4C and 4D present the comparison between the proposed method and the 2-D speckle tracking technique for simulated signals with 10 cm/s peak velocity. For the mild conditions the proposed method demonstrated lower error than the 2-D method for frame rates below 40 Hz, and similar performance above 40 Hz (Figure 4C). For the rough conditions the proposed method had significantly higher lower error compared to the 2-D technique below frame rates of 50 Hz.

### In-vivo simultaneous blood flow and myocardial velocity

**Figure 5:**
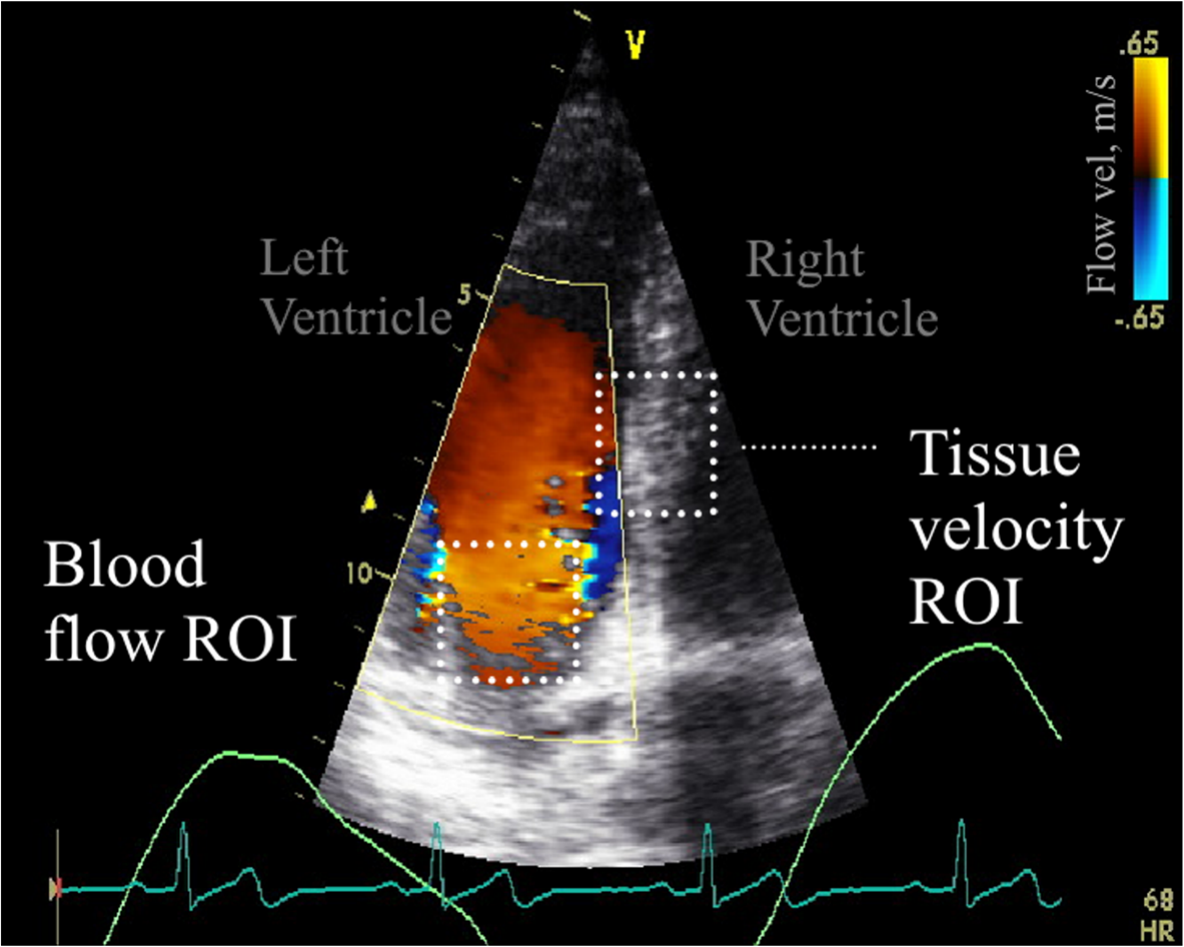
**Example of in-vivo Duplex mode ultrasound imaging of the heart from a healthy subject.** Image shows the filling of the left ventricle during diastole (red color is upward flow). The blood flow velocity (CDI mode) is color-coded on top of the grayscale tissue image (B-mode). The rectangular ROIs indicate the regions used for myocardial tissue velocity, and mitral blood flow velocity quantification. The frame rates of the CDI and B-mode image sequences were 100 and 33 Hz respectively. The ECG and respiration signals are visible at the bottom of the figure.

**Figure 6:**
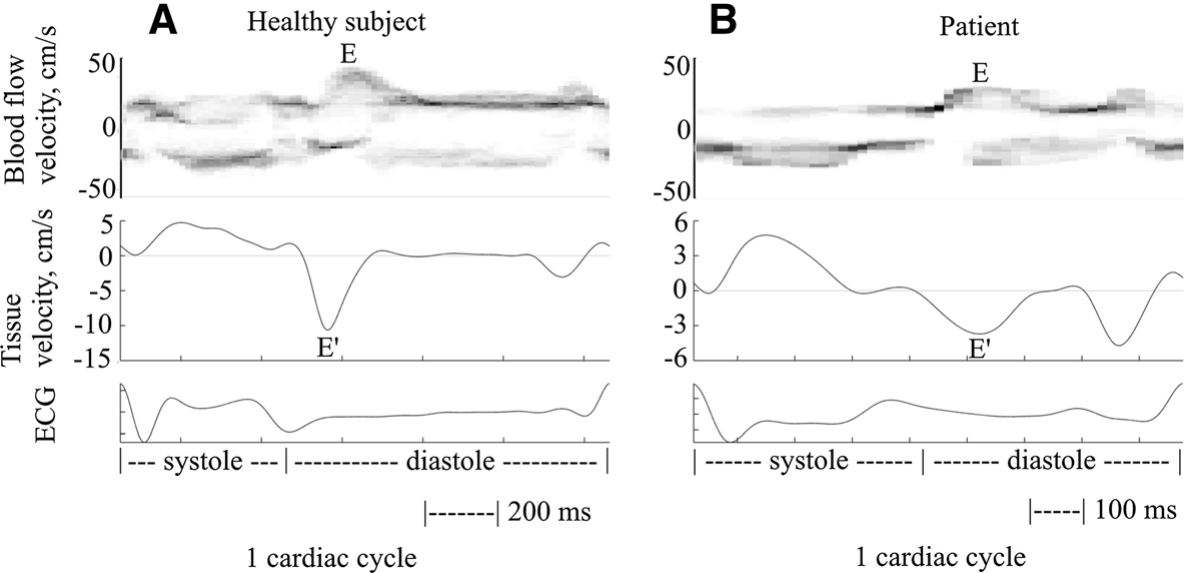
**Examples of quantification of simultaneous blood flow velocity (top) and myocardial motion (middle) during one cardiac cycle.** The corresponding electrocardiogram (ECG) can be seen in the bottom figure. The peak diastolic velocities are indicated as E and E’ for blood flow and myocardial tissue respectively. The peak of the tissue velocity preceded the peak of the blood flow in the healthy subject (left side), while the patient presented syncronized peaks (right side). The healthy subject also had higher velocities than the patient. These findings were expected.

Figure 5 shows an image from the Duplex mode ultrasound sector scan and its overlaid B-mode and CDI images for the healthy subject. The image was taken during the diastolic filling phase of the heart. Figure 6 demonstrates results from one cardiac cycle on simultaneous mitral blood flow velocity (top), estimated septum myocardial velocity (middle), and corresponding electrocardiogram (bottom). A clear difference in filling patterns was observed between the healthy subject and the patient. For the healthy subject the peak myocardial velocity preceded the peak blood flow in the diastolic phase, with E/E’ ≈ 5, and E/A ≈ 1.4. For the patient, the peak myocardial and blood flow velocities were synchronous, E/E’ ≈ 15, E/A ≈ 1.0, and the myocardial velocity was much lower than for the healthy subject.

## Discussion

This work presents a novel method for myocardial velocity quantification. The performance of the method was evaluated on image sequences at low frame rates, common with B-mode image sequences obtained from conventional Duplex mode ultrasound imaging. In addition, in-vivo simultaneous quantification of blood flow and myocardial tissue velocity was demonstrated.

### Myocardial velocity quantification

The performance of the proposed method shows that the performance was high at frame rates above 25 Hz, with a peak velocity error of less than 15% and low timing error (less than 10 ms). It should be stressed that the performance was evaluated on simulations of myocardial axial deformation in addition to both lateral and out of plane motion, and thus relatively rough conditions. The low timing error is likely attributed to the interpolation step of the velocity signal, and was also much lower than the theoretical frame rate limited error of 25 ms (at 25Hz).

The performance of the proposed method was comparable with the 2-D speckle tracking method under the mild conditions (best-case). However, for the rough conditions, the proposed method was superior to the 2-D method below frame rates of 50 Hz. This demonstrates the high robustness of the method at the low frame rates.

The peak velocity error was lower than, and timing error similar to what was found by Mårtensson et al. [7] studying the inter-system variations of tissue velocity imaging mode (TVI). They used a mechanical tissue phantom with pure axial displacement and found an error of 12.6% (range 0-34%) on myocardial peak diastolic velocity, and a timing error of 2.9 ms (range 0.6-10.7 ms). It should be stressed that they investigated much higher frame rates, between 92–168 Hz, as compared to ours at about 30 Hz.

The performance of the method was evaluated on synthetic B-mode image sequences derived using an in-house developed software. While some other studies using simulated ultrasound images have used the Field II simulation program [8], we did not use this due to the much higher computational complexity. Given the number of simulations required and degrees of freedom included in the myocardial motion and deformation, a comprehensive software for this purpose was developed. The myocardial tissue texture of the simulated B-mode images resembled the in-vivo images and also images from synthetic phantoms as used in [7].

### Simultaneous blood flow velocity and myocardial velocity

The results of the simultaneous blood flow and myocardial velocity from the two subjects were in accordance with what was expected: The myocardial motion preceded the mitral flow in the diastolic filling phase (blood is sucked into the ventricle due to a pressure gradient) in the healthy subject (Figure 6A). In the patient, there was no visible time lag between the blood flow and myocardial motion (Figure 6B) which is also typical for this group of patients. The indices of diastolic function (E/A, and E/E’) were similar to those reported in the literature for both subjects. Taken together the quantification of simultaneous myocardial motion and blood flow velocity was feasible based on Duplex mode imaging of a healthy and a patient.

One of the main goals in echocardiographic procedures is the estimation of cardiac pressures and pressure gradients (filling and relaxation pressures within the cavities of the heart). In particular, the ratio between the blood flow peak early diastolic velocities and myocardial early velocities (i.e., E/E’) is strongly correlated with the left ventricular filling pressure (LVFP) [9]. Li et al. [2] showed that this correlation is significantly improved when simultaneous E and E’ is used. Recently our group found that the temporal difference between isovolumic relaxation times (IVRT) measured from blood flow and myocardial motion (non simultaneous measurement) of early diastole was strongly correlated with LVFP [10]. Based on the low timing error of the proposed technique in the present work, a interesting future study is to study the relation between the IVRT and filling pressure when blood flow and myocardial velocity is measured in a simultaneous fashion.

The assessment of mitral flow velocity was based on the distribution of velocities within a ROI, which is similar to the typical representation in echocardiography [11]. However, it should be noted that the relatively low frame rate of blood flow velocity acquisition is known to cause lower velocities [12].

An important application of the proposed methodology could be in retrospective studies of simultaneous blood flow velocity and myocardial velocities analysing ultrasound scan stored in databases. In addition, the method may be applied for the study of variations in echocardiographic variables and indices over multiple cardiac cycles, due to the inherent motion robustness and relatively low computational complexity. Currently the authors are pursuing such studies to further investigate the coordination between blood flow velocity and myocardial motion in healthy subjects and in patients.

## Conclusions

This work presents a novel method for myocardial velocity quantification. The method demonstrated strong performance at frame rates above 25 Hz with a peak velocity error of less than 15%, and a peak timing error of less than 10ms. In addition, the method was superior to a traditional 2-D tracking method at frame rates below 50 Hz. In-vivo simultaneous quantification of blood flow and myocardial velocity was demonstrated based on conventional Duplex-mode ultrasound imaging and the proposed method. Simultaneous assessment of blood flow and myocardial velocity may allow detailed studies of the interaction between mechanical and hemodynamical processes within the heart. We propose that the method can be applied in both prospective as well as retrospective studies.

## Appendix A

### Synthetic tissue B-mode image sequence generation

Synthetic tissue echo mimicking pattern volumes were generated based on 3-D white noise, and each pixel value was taken as its 4^th^ power before the volume was 3-D low-pass filtered. The lateral resolution was decreased with increased depth. The parameters were set similar to those obtained using Duplex mode on the Vivid 7 (GE Medical, Horten, Norway), at 45 degrees field-of-view (FOV), resulting in a 3:1 ratio in frame rate between CDI and B-mode. The scan depth was 15 cm and the axial resolution was 0.2 mm/pixel. Synthetic B-mode image sector scans with 64 lines at 45 degrees FOV were generated by sampling image planes of the synthetic tissue volume at time stamps corresponding to a given frame rate (Figure 2A). Line-by-line acquisition was used to sample the sector scan lines. To simulate lateral and out-of-plane motion the 3-D tissue volume was displaced in the lateral and the out-of-plane direction, respectively, during the cardiac cycle, throughout the volume (5 mm peak displacement for both, with displacement waveform taken the same as the axial tissue velocity curve). The septum tissue was positioned in the center of the images with a 0.75 mm thickness, and left and right ventricles were set to zero intensity. This resulted in a realistic dynamic range. Finally, the B-mode scans were converted to 8-bit resolution. The axial tissue motion waveform was taken from a typical tissue velocity measurement [7] (Figure 2B), and was generated by compressing the axial tissue pattern, and lateral and out-of-plane tissue motion was generated by interpolating the 3-D tissue image according to the corresponding displacement at the time stamps. A random variation in temporal onset of the waveform was imposed as 0.5/framerate variation, and Gaussian white noise (10 dB, peak-to-peak) was added to the images.

## Abbreviations

ROI: Region-of-interest; B-mode: Brightness mode; CDI: Color doppler imaging; LVFP: Left-ventricular filling pressure; TVI: Tissue velocity imaging; SNR: Signal-to-noise ratio; FOV: Field-of-view.

## Competing interests

The authors declare that they have no competing interests.

## Authors’ contributions

CG: composed the manuscript, CG and KC: analysed the data and worked on the methods, CG and PL: proposed the idea, CG, JH, MH, PL: made the discussions. All authors read and approved the final manuscript.

## References

[B1] Sampath S Kimm JH Lederman RJ McVeigh ER Simultaneous imaging of myocardial motion and chamber blood flow with SPAMM n’ EGGS (Spatial Modulation of Magnetization with encoded gradients for gauging speed) J Magn Reson Imaging 2008 27 4 809 817 10.1002/jmri.21295 18383258 PMC2490799

[B2] Li C Zhang J Zhou C Huang L Tang H Rao L Will simultaneous measurement of E/e’ facilitate the non-invasive assessment of ventricular filling pressure in patients with non-valvular atrial fibrillation Eur J Echocard 2010 11 296 301 10.1093/ejechocard/jep218 20022868

[B3] Lou J Konofagou EE Imaging of wall motion coupled with blood flow velocity in the heart and vessels in vivo: A feasibility study Ultrasound in Med Biol 2011 37 980 995 10.1016/j.ultrasmedbio.2011.03.004 21546155 PMC4009734

[B4] Trahey GE Smith SW von Ramm OT Speckle-pattern correlation with lateral aperture translation: Experimental results and implications for spatial compounding IEEE Trans Ultrason Ferroelectr Freq Control 1986 32 257 264 18291782 10.1109/t-uffc.1986.26827

[B5] Bohs LN Trahey GE A novel method for angle independent ultrasonic imaging of blood flow and tissue motion IEEE Trans Biomed Eng 1991 38 280 286 10.1109/10.133210 2066142

[B6] Lin CH Lin MCJ Sun YN Ultrasound motion estimation using a hierarchical feature weighting algorithm Comp Med Imag Grap 2007 31 178 190 10.1016/j.compmedimag.2007.01.002 17317099

[B7] Mårtensson M Bjällmark A Brodin LÅ Evaluation of tissue Doppler-based velocity and deformation imaging: a phantom study of ultrasound systems Eur J Echocard 2011 10 467 476 10.1093/ejechocard/jer056 21565867

[B8] Jensen JA Field: A Program for Simulating Ultrasound Systems, Paper presented at the 10th Nordic-Baltic conference on biomedical imaging published in medical & biological engineering & computing Med Biol Eng Comput 1996 34 1 351 3538945858

[B9] Nagueh SF Middleton KJ Kopelen HA Zoghbi WA Quiñones MA Doppler tissue imaging: A noninvasive technique for evaluation of left ventricular relaxation and estimation of filling pressures J Am Coll Cariol 1997 30 1527 1533 10.1016/S0735-1097(97)00344-6 9362412

[B10] Lindqvist P Wikström G Waldenström A The use of E/Em and the time interval difference of isovolumic relaxation (TIVRT-IVRTm) in estimating left ventricular filling pressures Eur J Heart Fail 2008 10 490 497 10.1016/j.ejheart.2008.03.005 18406665

[B11] Szabo T Diagnostic ultrasound imaging: Inside-out (biomedical engineering) 2004 USA: Elsevier Academic Press

[B12] Sutherland GR Hatle L Claus P d’Hooge J Bijnens BH Doppler Myocardial Imaging – A Textbook 2006 Belgium: BSWK bvba, Scientific Consulting and Publishing- Hasselt

